# Innovative community-based vector control interventions for improved dengue and Chagas disease prevention in Latin America: introduction to the special issue

**DOI:** 10.1093/trstmh/tru176

**Published:** 2015-01-19

**Authors:** Johannes Sommerfeld, Axel Kroeger

**Affiliations:** a Special Programme for Research and Training in Tropical Diseases (TDR), World Health Organization (WHO), Geneva, Switzerland; b Liverpool School of Tropical Medicine, Liverpool, UK

**Keywords:** Chagas disease, Cluster randomized controlled trial, Community participation, Dengue, Latin America, Vector control

Dengue fever and Chagas disease are important public health problems in Latin America. Dengue is a re-emerging viral disease, mainly transmitted by *Aedes aegyptii* mosquitoes, leading to an increasing number of outbreaks notably in urban areas of the continent.^1,2^ Chagas disease, a parasitic disease transmitted by *Triatomine* bugs, is a major cause of morbidity and mortality among the continent's rural poor and persisting in different social-ecological settings.^3,4^ In spite of their epidemiological difference, both are vector-borne neglected tropical diseases (NTDs) for which primary prevention can currently mainly be achieved through vector control.^5^

In the case of dengue, routine vector control usually consists of source reduction strategies, including larviciding and/or insecticide space-spraying.^6^ However, vertically organized and insecticide-based vector control efforts often lack effectiveness and sustainability, and the need for community-based vector control strategies that include environmental management has been highlighted.^7–9^ With Chagas disease, routine interventions are usually based on insecticide spraying to eliminate household infestation. With a focus on domestic transmission, the peri-domestic transmission context is often neglected.

Current strategies for integrated vector management call for the adaptation of vector control interventions to local vector ecology, epidemiology and resources.^10^ Therefore, further insights relevant to specific ecosystems, into transmission dynamics and the possibility of intersectoral ecosystem management programs for dengue and Chagas disease prevention and control are urgently needed. This will play a crucial function in defining locally relevant and appropriate interventions with the prospects for sustainable control of vector populations.

This special issue reports findings of a research and capacity building program on innovative community-based vector control interventions for improved dengue and Chagas disease prevention in Latin America. The overall objective of the research initiative was to improve dengue and Chagas disease prevention by better understanding, through multi-level/multi-scale and trans-disciplinary analysis, ecosystem-related, biological and social (‘eco-bio-social’) determinants, and to develop and evaluate community-based public health interventions targeting dengue and Chagas disease vector habitats and delivered through intersectoral actions. The research program was a collaborative effort between the Special Programme for Research and Training in Tropical Diseases (TDR) and the Ecosystems and Human Health Program of the International Development Research Centre (IDRC).

## Methodology of the research

**Figure 1.**
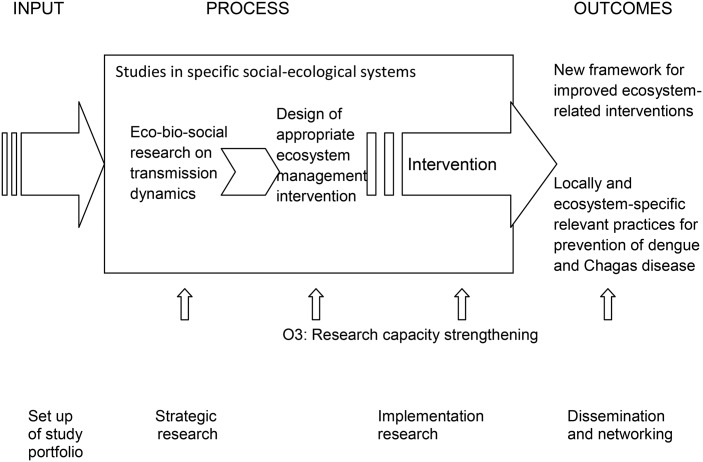
Conceptual framework of the research phases and objectives.

Eight multi-disciplinary research groups in seven countries of Latin America (Bolivia, Brazil, Colombia, Ecuador, Guatemala, Mexico, Uruguay) participated, forming a community-of-practice for ecohealth research on vector-borne diseases, with a focus on dengue in urban and peri-urban settings and on Chagas disease in rural settings. The overall initiative was based on the expectation that new scientific knowledge leads to improved dengue and Chagas disease prevention by informing and developing interventions in specific social-ecological settings (outcomes). Strategic research and implementation research, both accompanied by appropriate capacity strengthening efforts, were integral elements of the basic concept. The components of the research process are represented in Figure 1.

Based on a common core protocol and standardized data collection instruments, the research teams undertook an eco-bio-social situation analysis to characterize and map the ecosystem, vector ecology, the social context, including stakeholder environment, and community dynamics, including gender implications. A cross-site situation analysis on the five dengue studies was published earlier.^11^ The paper by Gürtler and Yadon^12^ in this special issue summarizes the situation analysis in the three Chagas disease research sites.

## Dengue research studies

The five studies on dengue were all carried out in urban contexts, in medium to large size urban agglomerations of Latin America: Acapulco/Mexico, Fortaleza/Brazil, Girardot/Colombia, Machala/Ecuador and Salto/Uruguay. In these urban settings, standard dengue vector control measures, routinely carried out (without or with limited community participation) by municipal or other governmental control services, usually consist of space-spraying and/or larviciding.

The study in Brazil^13^ took place in Fortaleza (Céara State) in the north-east of Brazil: a city particularly vulnerable to infestation with the dengue vector *A. aegyptii* due to its tropical climate, high demographic density and uncontrolled urbanization. The intervention was organized through urban communities by enhancing their participation in local environmental management actions focusing on discarded containers that were found in the situational analysis to be the most productive vector breeding sites in and around houses. In all intervention clusters, eco-health activities were organized, including the removal of discarded small recipients, cleaning of backyard areas and covering of large water containers on the ground and on the roof, but without the use of larvicides or insecticides.

The study in Colombia^14^ was conducted in Girardot, a municipality located 120 km from Bogotá on the right border of the Magdalena and Bogotá River. The intervention evaluated the efficacy, feasibility and cost-effectiveness of a combined approach of insecticide-treated window and door curtains (insecticide treated net [ITN] curtains) alone or in combination with covering the large productive water containers (>200 litre) with ITN materials. In the intervention clusters, long-lasting insecticide treated curtains on windows and water containers were deployed through local community networks and local entrepreneurs.

The Ecuador study^15^ was carried out in the city of Machala on the Pacific Coast of the country where *Aedes* indices are generally high and where there is an enhanced risk of dengue outbreaks. The intervention consisted of a comprehensive dengue elementary school education program, including classroom activities, practical skill development and application, and a ‘Clean Patio and Safe Container’ strategy focused on removing discarded or unused containers/materials from patios and brushing out and covering ground-level tanks.

The Mexico study^16^ was conducted in the city of Acapulco in the state of Guerrero on the Pacific coast of Mexico. The project deployed a novel intervention package based on targeted treatment of the most productive *Ae. aegypti* breeding sites and long-lasting insecticide treated screens permanently installed on windows and doors both at houses and schools. This was delivered through a community development approach working together with local authorities and small local enterprises.

The Uruguay study^17^ was carried out in the city of Salto, in northwestern Uruguay, on the border with Argentina, a city that currently does not face dengue transmission due to the marked seasonality of vector breeding. The intervention targeted productive container types and included household-based awareness and communication strategies, local media campaigns, roundtable discussions with doctors and health workers, and a partnership with the departmental health authorities.

## Chagas disease research studies

The projects on Chagas disease were carried out in rural research sites of Bolivia, Guatemala and Mexico, in areas where the socio-ecological setting is characterized by poverty and where social and environmental determinants lead to transmission in diverse domestic and peri-domestic contexts.

The study in Bolivia^18^ was carried out in poor rural communities of the Gran Chaco, inhabited by Guaraní Indians, and the Andean Dry Valley with Quechua Indians constituting the main population groups. The social-ecological setting is determined by domiciliated Chagas disease transmission through *Triatoma infestans.* The intervention consisted of housing improvement and the management of domestic animals, in particular those that enter houses: sleeping dogs, hens laying eggs, etc. and those that are used to sleeping outside but near to the house walls. The delivery strategy was based on strong community structures. In the Chaco Region, intervention support and ownership was ensured by ‘Capitanías’, the traditional political structure of the Guaraní indigenous people of the Bolivian Chaco. In the Valley Region, intervention support and ownership was provided by ‘Sindicatos’*,* the traditional political structure of the Guaraní indigenous people of the Bolivian Chaco. Sensitization of the community was done by community health agents (‘Agentes Comunitarios de Salud’) and through the organization of Chagas disease ‘health fairs’ (‘ferias de salud’).

The study in Guatemala^19^ was carried out in 18 communities (9 intervention, 9 control communities) in the municipality of Comapa, Department of Jutiapa, Eastern Guatemala. The transmission dynamics are determined by house infestation by non-domiciliated *T. dimidiata.* The intervention was focused on peridomestic animal management, with an emphasis on reducing rodents as reservoirs inside the house and integrated improved insecticide application, education regarding Chagas disease and risk factors, and a participatory rodent control program with mechanical trapping and environmental management to reduce rodent habitats.

The study in Mexico^20^ was carried out in three rural villages with a population of mainly Mayan descent in the State of Yucatan. The social-ecological setting is characterized by seasonal house infestation (during March to July between the wet and dry seasons) with limited colonization of non-domiciliated intrusive triatomines (*T. dimidiata).* A participatory action research approach led to a multi-stakeholder partnership built upon community and local government involvement, including a Chagas disease awareness campaign and cooperation between social workers and carpenters. The intervention consisted of housing improvement to prevent bug entry, including window screens mainly for bedroom windows, produced by local carpenters, and the management of the peridomestic environment, including cleaning and animal management.

## Conclusions from the studies

After completion of the situational analysis, the dengue research studies led to cluster randomized community trials in order to test the efficacy of different vector control approaches in terms of reducing vector densities and to analyze the feasibility and evaluate the sustainability of the community-based intervention strategies. In all sites, integrated, locally appropriate, control technologies for reducing both the immature and/or adult forms of the mosquitoes were used and implemented through community-based partnership models. The intervention tools ranged from insecticide treated window screens or curtains and water container covers (Mexico^16^, Colombia^14^) to non-chemical interventions, partnering communities and control program, to eliminate or cover the most productive vector breeding sites through waste management (Brazil^13^, Uruguay^17^) to educational efforts in schools and communities (Ecuador^15^).

The reduction of vector densities compared to control clusters was significant in Mexico, Brazil, Colombia and Ecuador (in spite of the ‘contamination’ of the control group with a large-scale larviciding program carried out concurrently); it was also present but not statistically significant in Uruguay (probably due to the small sample size). Building partnerships with communities and other stakeholders was crucial and the additional costs for health services were seen to be acceptable so that a scaling up program, financed by control programs or Ministries, is currently being initiated.

New strategies of empowering communities in contributing to garbage collection and recycling (Brazil, Uruguay) and of involving primary and secondary schools (Ecuador) to promote vector control activities at home and in the community were tested. They showed high acceptance rates, visibility and considerable impact on vector densities. Strengthening community involvement and establishing prolonged interaction of community representatives with control services, municipalities and other public actors were shown to be time consuming and costly at the beginning, but rewarding during the process and with excellent potentials for sustainability (Sierra E. A., unpublished data).

The earlier findings that routinely used ‘larval surveys’ to determine the presence or absence of dengue vectors should be complemented by annual or biannual ‘pupal productivity surveys’^21,22^ during the wet season in order to identify productive container types for targeted interventions could be confirmed. Likewise, the characteristics of water containers producing most of dengue vectors could be identified: outdoor, rainwater filled, not protected, untreated and in shaded areas. For such targeted interventions, new and innovative dengue vector control tools have been developed and tested and they have been shown to have a decisive impact on the vector populations. In particular, (1) window and door screens (with insecticide treated netting material) in a fixed aluminium frame adapted to local window types (Mexico), and (2) water container covers using a similar design but with a flexible opening (Colombia); both devices were highly appreciated by the population and vector control services. This innovative approach is attractive for dengue control services in several countries (Brazil, Colombia, Mexico, Uruguay). Policy makers and practitioners were an active part of the research initiative and committed to scaling up the interventions at city levels in four sites (Brazil, Colombia, Mexico, Uruguay).

The three Chagas disease studies provide evidence on innovative interventions that go beyond routine indoor residual spraying of insecticides against domestic vectors of Chagas disease by national control services. These studies renewed the attention to interventions that address social and environmental determinants (e.g., improvement of housing conditions, notably in the Bolivia study) through social participation. Targeting the peri-domestic transmission context (Mexico) and a focus on peridomestic animal management (Guatemala) were innovative features of the research portfolio on Chagas disease. Both domestic and peri-domestic transmission contexts can be addressed through participatory multi-stakeholder processes, which combine routine indoor residual spraying with improvement of housing conditions, management of domestic and peri-domestic animals, and general environmental hygiene.

Thus, the research initiatives featured in this special issue, have generated evidence on the feasibility and impact of innovative, locally adapted approaches to vector control of two important vector-borne diseases in Latin America, dengue and Chagas disease, through community-based partnership models and through environmental management approaches.
